# Supplementary material for: Dual process impairments in reinforcement learning and working memory systems underlie learning deficits in physiological anxiety
Source: PLoS Comput Biol. 2025 Sep 26;21(9):e1012872. doi: 10.1371/journal.pcbi.1012872 (PMC12500139; doi:10.1371/journal.pcbi.1012872)
Supplement: S1 Text — (PDF) [file pcbi.1012872.s001.pdf]

a)

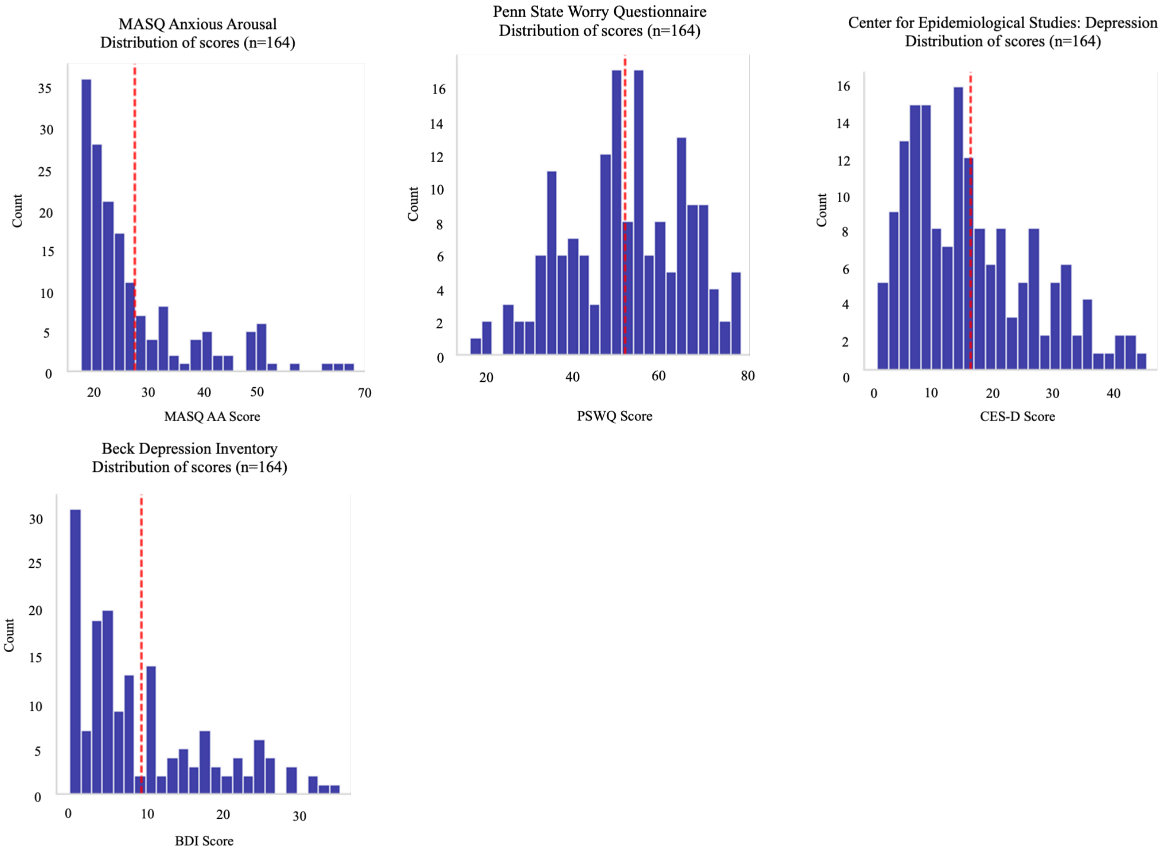

b)

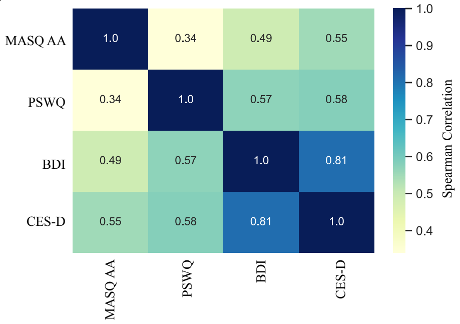

Figure A. Distributions of questionnaire metrics in final participant group (n=164)
a) Histograms showing group distribution of scores for MASQ AA, PSWQ, CES-D, and BDI-II as labeled. Red dashed vertical line denotes group mean score. b) Correlation between subscale scores across participants.

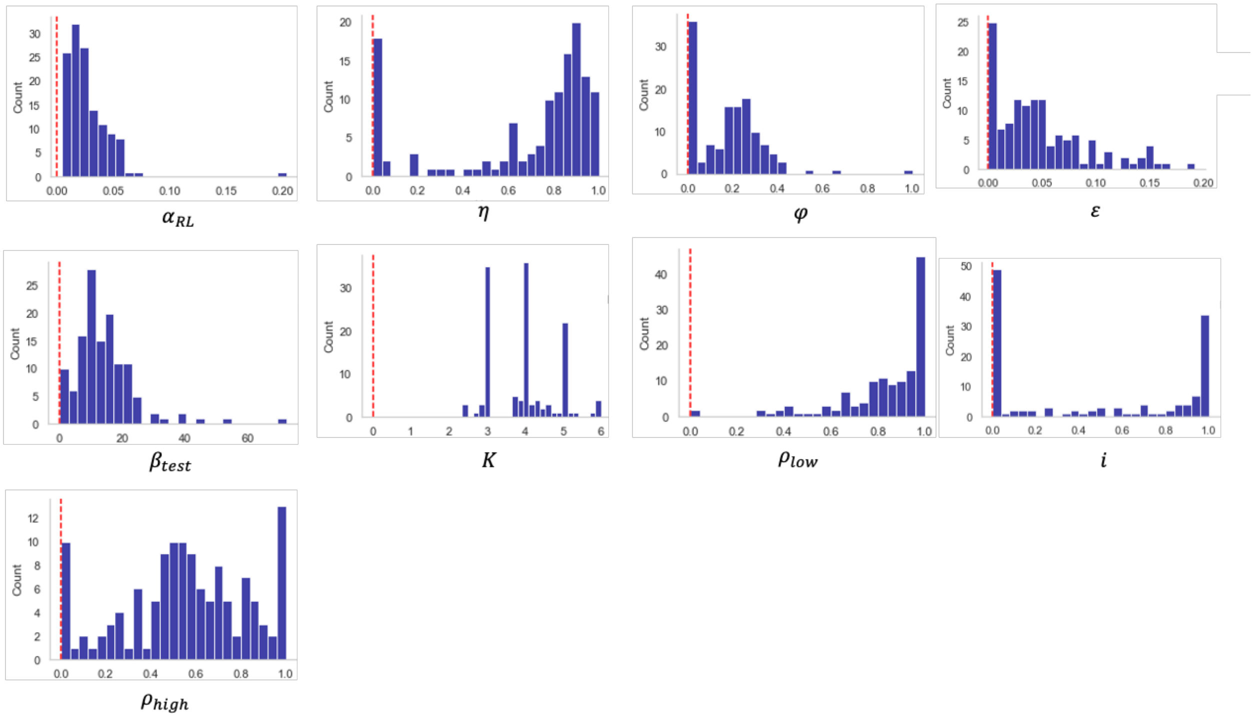

Figure B. Parameter value distributions: Model #5
Histograms showing group distribution of parameter values for winning model (Model #5) as labeled.

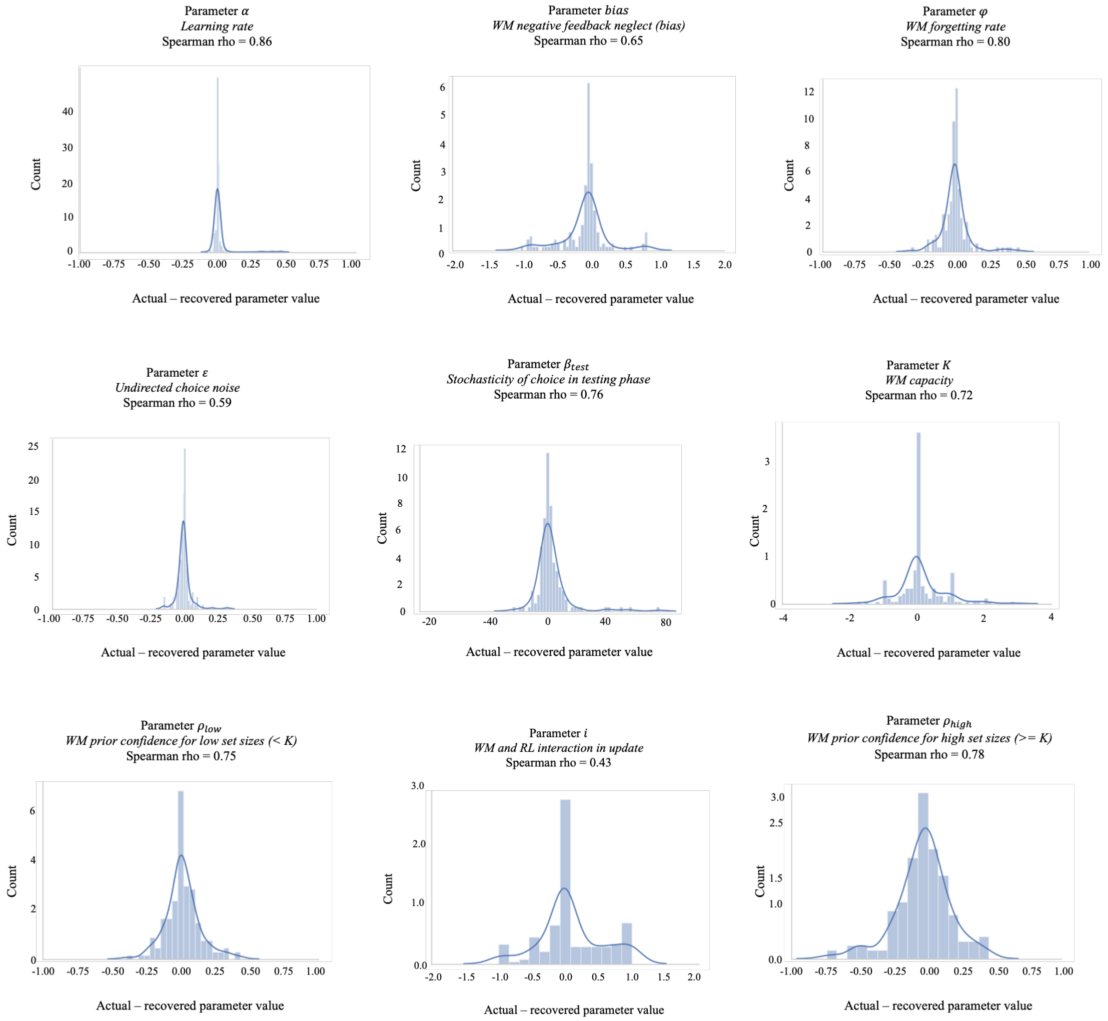

Figure C: Parameter recovery analyses: Model #5

For each parameter in the winning model (Model #5), the distribution of differences between actual and recovered parameter values is shown. The correlation between actual and recovered parameter values (as measured with Spearman rho) is denoted in text above each plot. See Methods for details.

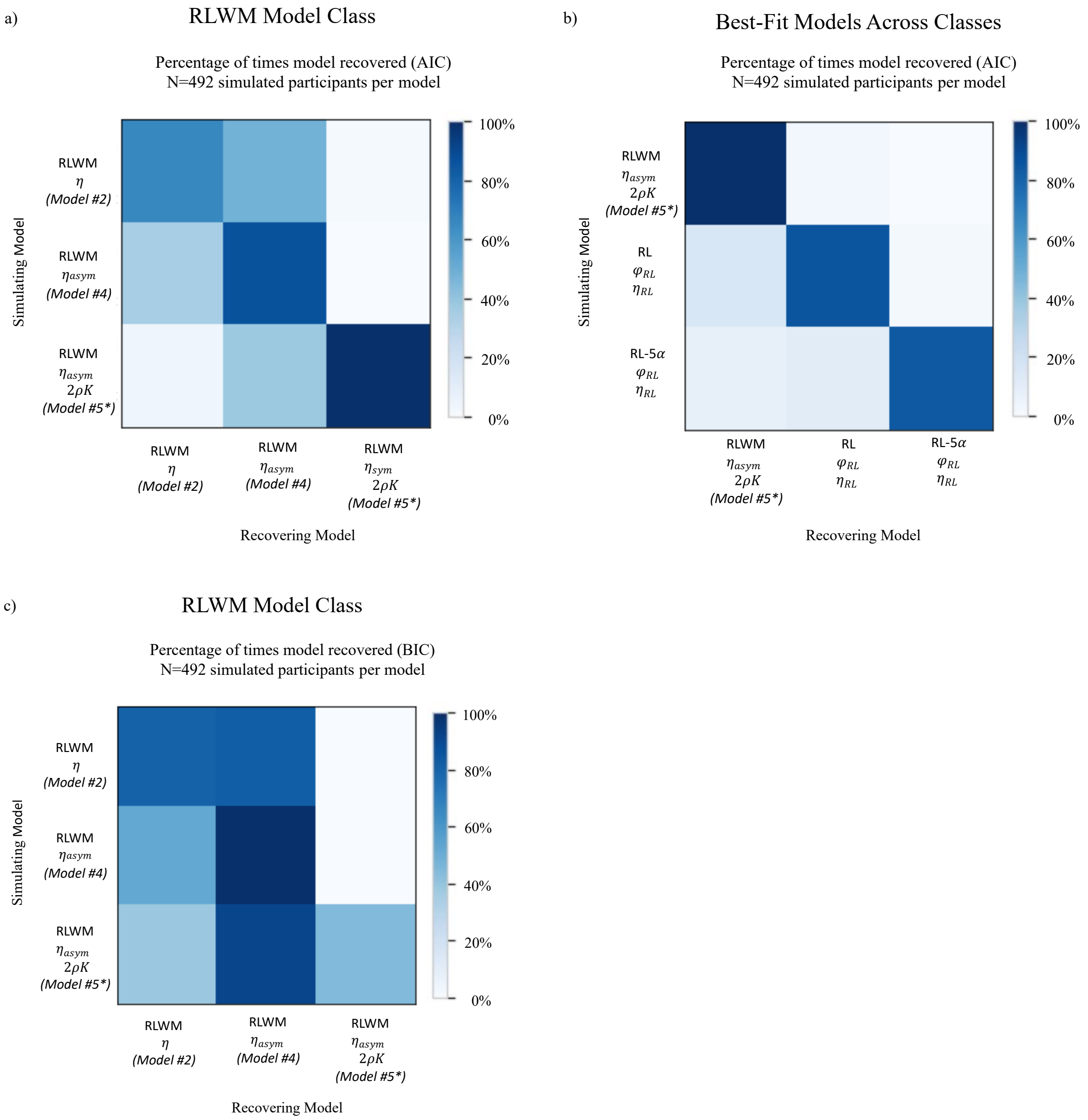

** Indicates winning model for main analyses.*

Figure D: Model recovery analysis

Comparison of frequency with which the generative model provided the best fit to the data. a) Model recovery analysis performed using AIC between main analysis winning model (Model #5) used for hypothesis testing; main analysis second-best fit model (Model #4) used for robustness testing; and bias-parameter model (Model #2) for representative recovery analyses. b) Model recovery analysis performed using AIC between main analysis winning model (Model #5); best-fit RL-only model ($RL\_\varphi_{RL}\_\eta_{RL}$); and best-fit RL-only 5α model ($RL\_5\alpha\_\varphi_{RL}\_\eta_{RL}$). See Methods for further model details. c) Model recovery analysis as in a) using Bayesian Information Criteria (BIC) as metric of model fit. Note that BIC over-penalizes RLWM models, with more complex generative processes being incorrectly fit by simpler models.

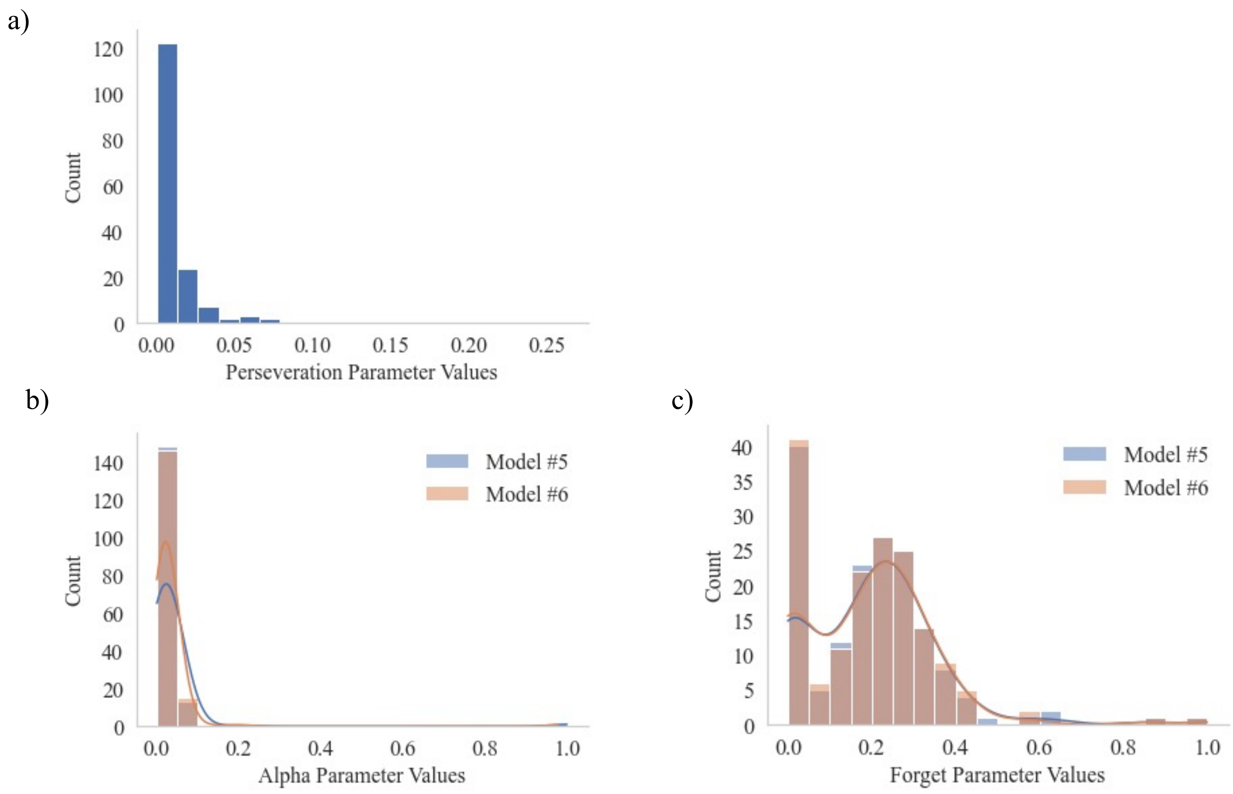

Figure E: Robustness check of winning model (Model #5) against choice kernel model variant (Model #6)
a) Perseveration parameter values from Model #6 reveal minimal perseveration across participants. b) Learning rate parameter values for Model #5 are not significantly different from values for Model #6 (Man-Whitney-U test: U-statistic=13,796.0, p=0.686). c) WM decay parameter values for Model #5 were not significantly different from values for Model #6 (Man-Whitney-U test: U-statistic=13,669.0, p=0.797).

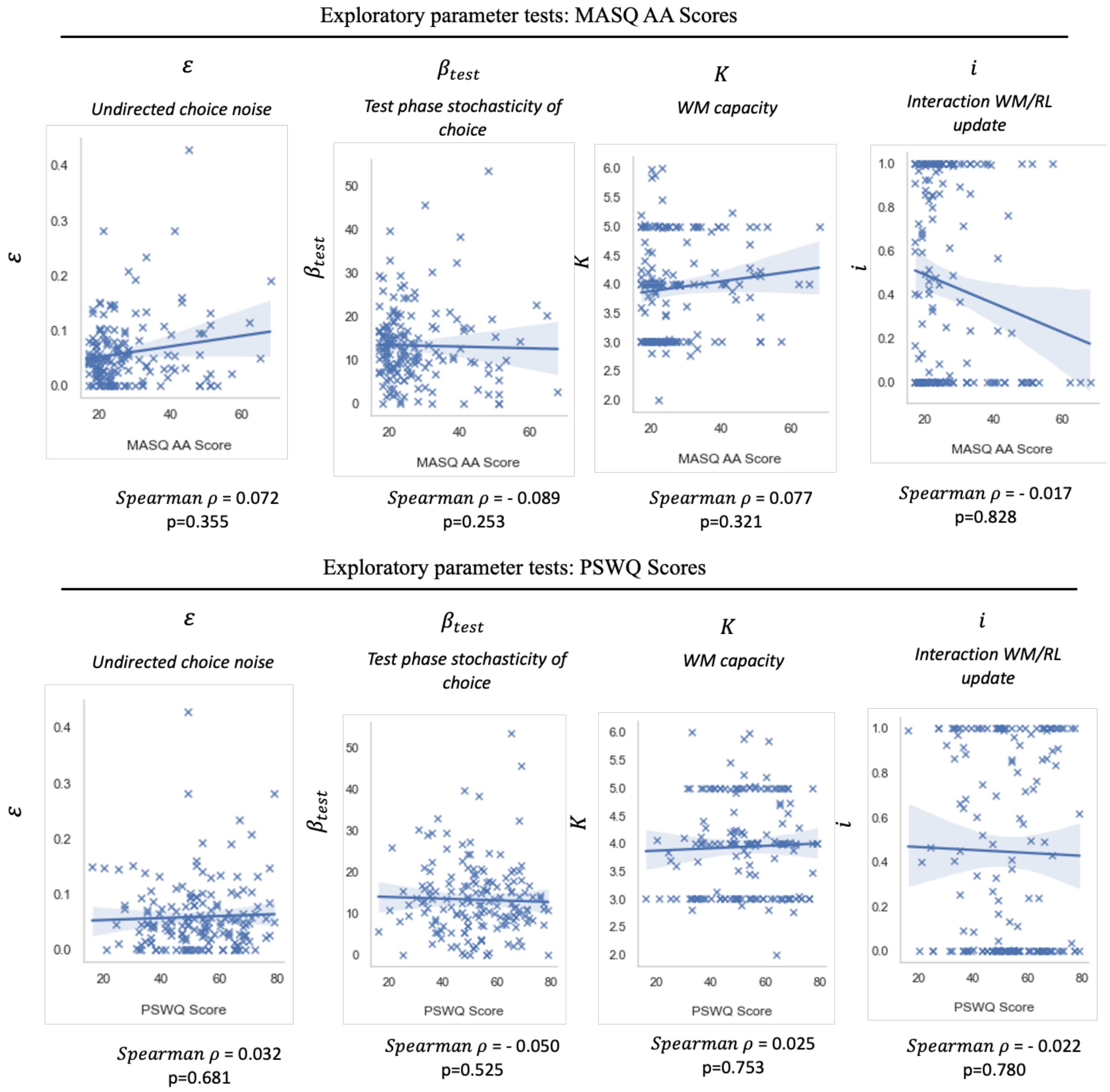

Figure F: Exploratory parameter testing Model #5 for MASQ AA and PSWQ
Exploratory testing of Model #5 parameters with MASQ AA and PSWQ scores. p-values uncorrected for multiple comparisons/family-wise error.

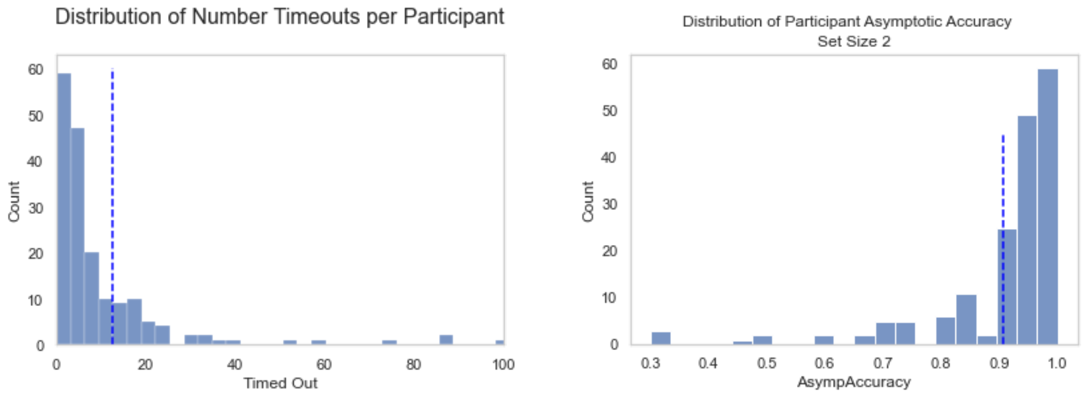

Figure G: Exclusion statistics
Distribution of number of timeouts per participant prior to timeout-based exclusions (left) and distribution of participant asymptotic accuracy in learning over last 5 stimulus presentations at set size 2 (right). Dashed blue lines denote mean. Exclusion criterion was 2SD from mean (mean + 2sd for timeouts; mean – 2sd for set size 2 performance).

Supplemental Analyses

Test of winning RLWM model (Model #5) robustness to bias when choice kernel is included

Recent work (43) has established that in some (but not all) RL data sets, the absence of a choice perseveration kernel in the associated RL model can bias estimation of negative feedback neglect. To ensure that our winning model, which did not include a choice perseveration kernel, did not result in biased parameter estimation relevant to results, we tested the fit of the winning model (Model #5) if it was modified to include a choice kernel, allowing for a parametrically decaying influence of past choices on the current choice (Model #6; see Methods). Model #6 did not fit the data as well as Model #5 based on AIC. In addition to worse model fit based on AIC, values for the parameter quantifying perseveration were very close to 0 (see Figure E (a) in S1 Text), and neither of the key parameters of interest from Model #5 (namely those for which there were significant findings: learning rate α and WM decay φ_{WM}) differed significantly in value between Model #5 and Model #6 (see Figure E (b,c) in S1 Text).

Test of robustness of results in second-best-fitting RLWM model (Model #4)

As a robustness check across models, we tested the significant findings between MASQ AA subscale scores and model parameters in the second-closest winning model (Model #4), a nested version of the winning model with fewer parameters (notably, Model #4 did not include separate working memory confidence parameters for low versus high set sizes). Confirmatory test p-values were corrected using the Bonferroni statistical correction for 3 comparisons. As in Model #5, higher MASQ AA subscale scores were significantly related to reduced learning rate parameter (rho(162) = -0.270, FWE-corrected p = 0.004) and higher rate of working memory decay

(rho(162) = 0.245, FWE-corrected p = 0.001) in Model #4. We note that if we had performed initial hypothesis testing in Model #4 instead of Model #5, we would have performed 8 total comparisons (4 model parameters from initial hypothesis testing across 2 trait measures). The relationship of MASQ AA scores with both learning rate and with rate of working memory decay in Model #4 also survive this more conservative metric of multiple comparisons (learning rate: 8-comparison FWE-corrected p = 0.003; working memory decay: 8-comparison FWE-corrected p = 0.012).

Behavior: Reaction Time

Mean reaction time (RT) across correct learning trials was 599.5ms, and reaction times increased as a function of set size during learning (Kruskal-Wallis statistic(5) = 11308.01, p=0.0). There was a weakly trending effect of PSWQ score on longer overall mean learning reaction time (Spearman rho(162) = 0.139, p = 0.076), but no effect of MASQ AA subscale score on overall mean reaction time (Spearman rho(162) = -0.086, p = 0.276) across set sizes. A nonparametric test of correlation at each set size revealed that higher MASQ AA subscale scores were associated with a significantly shorter reaction time for set size = 5 only (Spearman rho(162) = -0.182, p = 0.02), but found no other significant relationships between MASQ AA or PSWQ scores and learning phase RTs in other set sizes. Scores on the MASQ AA subscale were significantly related to RT set size slope in learning (Spearman rho(162) = 0.178, p = 0.023), suggesting an increased sensitivity of RT to set size increases for higher MASQ AA subscale scores. PSWQ scores were not related to RT set size slope in learning (Spearman rho(162) = -0.017, p = 0.827).

Mean RT across correct testing phase trials was 557.8ms, and was significantly lower than learning phase RT for correct trials (Mann-Whitney U statistic(1) = 617273595.0, p=1.92e-72). There was no significant effect of set size on testing phase RT (Kruskal-Wallis statistic(5) = 1.36, p=0.851). PSWQ scores were not significantly correlated with longer overall testing phase RT (Spearman rho(162) = 0.118, p = 0.131), nor were MASQ AA subscale scores (Spearman rho(162) = -0.086, p = 0.273). A nonparametric test of correlation at each set size revealed that higher PSWQ scores were associated with significantly longer reaction times for set size = 4 (Spearman rho = 0.224, p = 0.01) and set size = 5 (Spearman rho(162) = 0.174, p = 0.047) only. There were no other significant relationships between MASQ AA or PSWQ scores and testing phase RTs by set size. RT set size slope in testing was not significantly related to either MASQ AA subscale scores (Spearman rho(162) = -0.038, p = 0.628) or PSWQ scores (Spearman rho(162) = -0.042, p = 0.595).

Behavior: Additional statistics for PSWQ with learning and testing performance

A repeated measures ANCOVA with within-subject factor of set size, covariate of z-scored PSWQ scores, and dependent variable of mean learning performance revealed no significant main effect of PSWQ (between-subjects PSWQ F(1,162) = 1.200, p = 0.275) and no significant interaction of PSWQ and set size (within-subject set size x PSWQ effect with Greenhouse-Geisser correction F(3.415, 553.151) = 0.839, p = 0.485). There was a main within-subject effect of set size (within-subject set size effect with Greenhouse-Geisser correction F(3.415, 553.151) = 64.945, p < 0.001).

A repeated measures ANCOVA with within-subject factor of set size, covariate of z-scored PSWQ scores, and dependent variable of mean testing performance revealed no significant main effect of

PSWQ (between-subjects PSWQ $F(1,162) = = 0.672$, $p = 0.414$) or interaction between set-size and PSWQ (within-subject set size x PSWQ effect with Greenhouse-Geisser correction $F(3.729, 604.140) = 1.321$, $p = 0.262$).

We additionally tested the relationship between PSWQ scores and drop between asymptotic accuracy over last 3 trials during learning and overall performance during testing. A repeated-measures ANCOVA with within-subject factor of set size, covariate of z-scored PSWQ scores, and dependent variable of drop in performance between asymptotic final 3 trial learning performance and mean overall testing performance revealed no main effect of PSWQ scores (between-subjects PSWQ $F(1,162) = 0.429$, $p = 0.514$) or interaction between set size and PSWQ (within-subject set size x PSWQ effect $F(4,648) = 1.218$, $p = 0.302$). There was a main effect of set size (within-subjects set size effect $F(4,648) = 9.005$, $p < 0.001$).

Exploratory analyses in winning RLWM model (Model #5)

Following specific hypothesis testing, we performed an exploratory analysis of the (FWE-uncorrected) relationships between all model parameters and scores on the two measures of anxiety used for hypothesis testing above (MASQ AA and PSWQ) as well as two measures of trait depression (the CES-D scale and the Beck Depression Inventory (BDI) II). There were no significant correlations between MASQ AA, PSWQ, CESD, or BDI scores and any model parameters other than those reported for MASQ AA in the main analysis.
